# Construction and comprehensive characterization of an *Ec*LDCc-CatIB set—varying linkers and aggregation inducing tags

**DOI:** 10.1186/s12934-021-01539-w

**Published:** 2021-02-17

**Authors:** Kira Küsters, Martina Pohl, Ulrich Krauss, Gizem Ölçücü, Sandor Albert, Karl-Erich Jaeger, Wolfgang Wiechert, Marco Oldiges

**Affiliations:** 1 Institute of Bio- and Geosciences IBG-1: Biotechnology, Forschungszentrum Jülich GmbH, 52425 Jülich, Germany; 2 Institute of Biotechnology, RWTH Aachen University, 52074 Aachen, Germany; 3 Institute of Molecular Enzyme Technology, Heinrich-Heine-Universität Düsseldorf, Forschungszentrum Jülich GmbH, 52425 Jülich, Germany; 4 Faculty of Biotechnology, University of Applied Sciences Mannheim, 68163 Mannheim, Germany; 5 Computational Systems Biotechnology (AVT.CSB), RWTH Aachen University, 52074 Aachen, Germany

**Keywords:** Catalytically active inclusion bodies, Immobilization, Protein aggregates, Protein engineering, Downstream processing, Microscopic analysis, Enzymes

## Abstract

**Background:**

In recent years, the production of inclusion bodies that retained substantial catalytic activity was demonstrated. These catalytically active inclusion bodies (CatIBs) were formed by genetic fusion of an aggregation inducing tag to a gene of interest via short linker polypeptides and overproduction of the resulting gene fusion in *Escherichia coli*. The resulting CatIBs are known for their high stability, easy and cost efficient production, and recyclability and thus provide an interesting alternative to conventionally immobilized enzymes.

**Results:**

Here, we present the construction and characterization of a CatIB set of the lysine decarboxylase from *Escherichia coli* (*Ec*LDCc), constructed *via* Golden Gate Assembly. A total of ten *Ec*LDCc variants consisting of combinations of two linker and five aggregation inducing tag sequences were generated. A flexible Serine/Glycine (SG)- as well as a rigid Proline/Threonine (PT)-Linker were tested in combination with the artificial peptides (18AWT, L6KD and GFIL8) or the coiled-coil domains (TDoT and 3HAMP) as aggregation inducing tags. The linkers were fused to the C-terminus of the *Ec*LDCc to form a linkage between the enzyme and the aggregation inducing tags. Comprehensive morphology and enzymatic activity analyses were performed for the ten *Ec*LDCc-CatIB variants and a wild type *Ec*LDCc control to identify the CatIB variant with the highest activity for the decarboxylation of l-lysine to 1,5-diaminopentane. Interestingly, all of the CatIB variants possessed at least some activity, whilst most of the combinations with the rigid PT-Linker showed the highest conversion rates. *Ec*LDCc-PT-L6KD was identified as the best of all variants allowing a volumetric productivity of 457 g L^− 1^ d^− 1^ and a specific volumetric productivity of 256 g L^− 1^ d^− 1^ g_CatIB_^−1^. Noteworthy, wild type *Ec*LDCc, without specific aggregation inducing tags, also partially formed CatIBs, which, however showed lower activity compared to most of the newly constructed CatIB variants (volumetric productivity: 219 g L^− 1^ d^− 1^, specific volumetric activity: 106 g L^− 1^ d^− 1^ g_CatIB_^− 1^). Furthermore, we demonstrate that microscopic analysis can serve as a tool to find CatIB producing strains and thus allow for prescreening at an early stage to save time and resources.

**Conclusions:**

Our results clearly show that the choice of linker and aggregation inducing tag has a strong influence on the morphology and the enzymatic activity of the CatIBs. Strikingly, the linker had the most pronounced influence on these characteristics.

## Background

Enzymes produced by microbial systems becoming increasingly important, e.g., for the sustainable production of platform chemicals and bio-based polymers [1–4]. Due to their advantages, like heat resistance, tensile strength and electrical insulation, polyamides are interesting for diverse applications in the electrical, automotive and textile industry as well as for consumer articles and in the medical sector [5]. One successful example of a biotechnologically produced precursor for a bio-based polyamide is 1,5-diaminopentane (DAP). Together with dicarbonic acids like sebacic acid, this C5 diamine building block is used to build up polyamides (PA). The resulting PA 5.10 (5: 1,5-diaminopentane (C5); 10: sebacic acid (C10)) shows comparable or even better material properties compared to the widely used petroleum-based polyamide PA 6 (6: caprolactam (C6)) [6]. DAP can be biotechnologically produced from l-lysine by enzymatic decarboxylation through the constitutive lysine decarboxylase (LDCc) [7] or the acid-induced variant CadA [8] from *Escherichia coli*. Both enzymes use pyridoxal 5’-phosphate (PLP) as a cofactor. Kloss and coworkers showed a workflow where *Corynebacterium glutamicum* was used to produce l-lysine from glucose. The l-lysine was then enzymatically decarboxylated to yield DAP by the native *Ec*LDCc, which was overproduced in *E. coli*, [9].

In biocatalysis such enzymatic conversions are often performed using whole cell systems or purified soluble enzymes, whereas Kloss et al. used catalytically active inclusion bodies (CatIBs) of the *Ec*LDCc to decarboxylate l-lysine to DAP [7–9]. Even though purified enzymes can be used to catalyze reactions with high activities, their application requires respective costly and laborious downstream processing and purification procedures [10–13]. Moreover, the recycling of purified enzymes from biotransformations is more difficult to achieve and usually requires application of membrane separation, membrane reactor application or particle-based immobilization strategies [14–16].

To simplify the reusability and enhance the stability, immobilization of enzymes is often used, resulting in macromolecular or heterogeneous catalysts [17–19]. Common immobilization strategies rely first on the production of the soluble enzyme in an expression host. Subsequently, purification and lastly immobilization by e.g. covalent binding, cross-linking, binding the enzyme to carrier or entrapment of the enzyme is performed [20, 21]. However, enzyme immobilization often comes at the expense of overall activity of the immobilized enzyme preparation. This could be either due to reduced activity of the enzyme or the reduced mass transfer of reaction partners within the immobilized protein matrix.

A simpler and more cost efficient strategy is the use of CatIBs. For a long time, inclusion bodies (IBs) were regarded as inactive and misfolded protein aggregates. However studies revealed that catalytically active inclusion bodies with a reasonable residual activity can be produced by fusion of an enzyme of interest with a linker, composed of a few amino acids, and an aggregation inducing tag. Two recent reviews provided a comprehensive overview over suitable linker and aggregation inducing tags that have been successfully used for CatIB formation [22, 23]. The aggregation inducing tags in this study are the coiled coil domain of the cell-surface protein tetrabrachion from *Staphylothermus marinus* (TDoT) as well as the dimeric coiled coil domain from *Pseudomonas aeruginosa* (3HAMP) [9, 24, 25]. Moreover, the aggregation inducing tag properties of three artificial peptides, a small surfactant-like L6KD peptide, an amphipathic α-helical peptide (18AWT) and a hydrophobic self-assembling peptide (GFIL8) were also analyzed [26–28]. In contrast to other enzyme formulations CatIBs possess many advantages, such as (i) simple purification, (ii) high stability, (iii) easy long-term storage, (iv) carrier-free, biodegradable and biologically produced immobilization technology, (v) reusability as well as they are considered as (vi) essentially GMO-free after separation from the producer cells [9, 22, 24, 29, 30].

However, at present, there is only limited knowledge that would allow predicting a successful combination of a target enzyme, a linker and an aggregation inducing tag. For example, CatIB formation was tested for different enzymes, such as the benzaldehyde lyase from *Pseudomonas fluorescens*, the alcohol dehydrogenases from *Ralstonia sp*. and *Lactobacillus brevis* as well as for the *Bacillus subtilis* lipase A. Here, CatIBs with varying residual activity were formed, depending on the selected aggregation inducing tag [24, 26–28]. Thus, to realize efficient CatIB formation, many different variations need to be generated and tested to find the best-performing combination of target enzyme, linker and aggregation inducing tags. So far, most of the CatIBs described in literature were generated using traditional cloning methods, which limits the fast access to a CatIB library [24–28;31–35].

One option to create such a library is Golden Gate Assembly, which relies on Type IIS restriction enzymes. These enzymes cleave the DNA outside their recognition site, allowing the generation of specific desired overhangs. The generated four-nucleotide overhang can only be ligated to the matching DNA overhang from the following fragment. Because restriction digest and ligation happen at the same time, the reaction takes place in a so-called “one-pot setup” [36]. Due to these features of the Golden Gate Assembly, three different DNA elements can be assembled in an effortless manner, thereby allowing the high-throughput generation of large CatIB libraries. This speeds up the search for the best performing CatIB-construct, while at the same time allowing the generation of large datasets useful for understanding structure/function relationships between the CatIB constituting modules. This in turn, could enable a more rational prediction of suitable elements for CatIB formation in the future.

Here, we report the generation and characterization of an *Ec*LDCc-CatIB set, generated *via* Golden Gate Assembly. A combination of two different linkers and five different aggregation inducing tags were fused to the C-terminus of the *Ec*LDCc resulting in ten different combinations. The resulting CatIB variants were analyzed comprehensively with regard to CatIB and cell morphology as well as activity of the CatIBs, proving that the linker and aggregation inducing tag revealed a strong influence on these features.

## Results and discussion

### Production of CatIBs and microscopic analysis

**Fig. 1:**
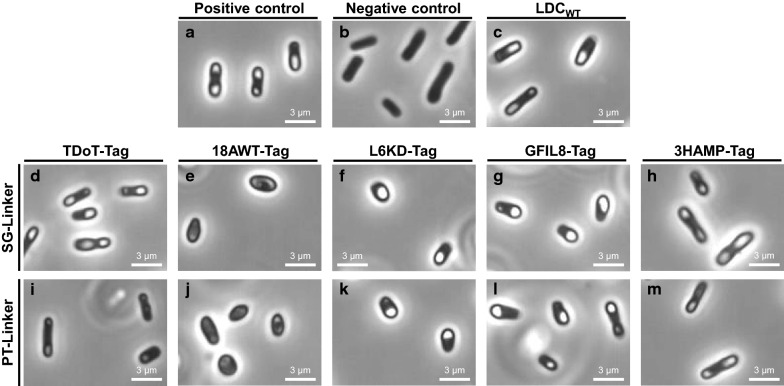
Microscopic images of cells producing different *Ec*LDCc-CatIB variants and control cells at 1000-fold magnification. Cultivation was performed for 3 h at 37 °C and 69 h at 15 °C in M9-AI medium. **a** Positive control: *E. coli* BL21(DE3) with *Ec*LDCc-Xa-SG-TDoT [9], **b** Negative control: *E. coli* BL21(DE3) with pET28a, **c** wild type control: *E. coli* BL21(DE3) with *Ec*LDCc **d** *Ec*LDCc-SG-TDoT, **e** *Ec*LDCc-SG-18AWT, **f** *Ec*LDCc-SG-L6KD, **g** *Ec*LDCc-SG-GFIL8, **h** *Ec*LDCc-SG-3HAMP, **i** *Ec*LDCc-PT-TDoT, **j** *Ec*LDCc-PT-18AWT, **k** *Ec*LDCc-PT-L6KD, **l** *Ec*LDCc-PT-GFIL8, **m** *Ec*LDCc-PT-3HAMP

The lysine decarboxylase of *E. coli* (*Ec*LDCc; EC 4.1.1.18), was C-terminally fused with one of two linkers (a flexible SG- or a rigid PT-Linker) as well as one aggregation inducing tag out of the set of five, TDoT, 18AWT, L6KD, GFIL8 and 3HAMP [9, 26–28, 37]. *Ec*LDCc shows a decameric quaternary structure with the N-terminus being buried at the inner side of the decameric ring-like structure. Therefore, the linkers and the aggregation inducing tags were fused to the C-terminus of the *Ec*LDCc. The ten different CatIB variants were produced in *E. coli* BL21(DE3) using M9 autoinduction medium (See Additional file 1: Table S2). The formation of *Ec*LDCc-CatIBs in this host was verified using phase contrast microscopy with a 1000-fold magnification (see Methods). CatIBs appear as white refractive particles or granule-like structures at the cell poles (Fig. 1), which is typical for IBs [38].

Microscopic analyses of all strains were performed to test if the CatIB variants produce CatIBs with different morphologies, and if CatIB production, in turn, affects the morphology of the producing cells. The positive control (*Ec*LDCc-Xa-SG-TDoT, more data about this variant was published by Kloss et al. [9]) formed dense CatIBs, whereas the negative control (empty pET28a vector) did not show any detectable IBs. Contrary to expectation, the wild type *Ec*LDCc control also formed IBs, which usually is a consequence of strong gene expression and often results in complete activity loss of the enzyme [39–41].

Microscopic analysis of the *Ec*LDCc-CatIB variants revealed that different combinations of linkers and aggregation inducing tags led to different shapes of cells and IBs and morphologies. IBs were found very similar with both linkers and with the aggregation inducing tags L6KD, GFIL8, and 3HAMP. In contrast, the TDoT variant formed large and dense IBs in combination with the flexible SG-Linker only, while the respective *Ec*LDCc-PT-TDoT generated only small and diffuse IB structures and only 61 % of the cells carrying this construct produced IBs at all (Table 1). By contrast, the other CatIB producing variants showed that 71 % to 88 % of the cells produced CatIBs with a mean number of CatIBs per cell in the range of 1.18 for *Ec*LDCc-PT-L6KD up to 1.83 for *Ec*LDCc-PT-3HAMP. Strikingly, *E. coli* cells carrying constructs with the aggregation inducing tag 18AWT did not show any visible dense IBs at all. This might be because this tag is known to show a tendency to bind to the cell membrane [28], which could be responsible for the abnormal shape of the cells. Because of the absence of dense, refractive IBs, no further CatIB morphology analysis could be performed for these variants (Fig. 1). Absence of IBs in phase contrast microscopic images of the variants with aggregation inducing tag 18AWT, do not necessarily mean that there were no IBs at all. Small shaped or membrane associated IBs might had not detected, although IBs or even CatIBs could have been present. In this sense, different picture generating methods with higher precision, such as scanning electron microscopy could provide better insights.

**Fig. 2:**
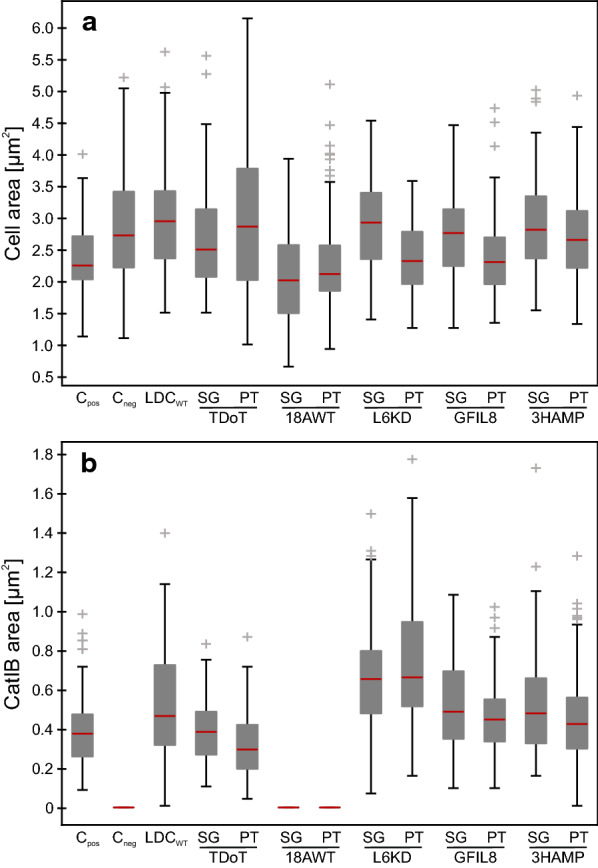
Size distribution of **a** *Ec*LDCc-CatIB producer strains and **b** *Ec*LDCc-CatIBs. The median (red), the 25 and 75 % Quartiles (grey box), the 5 % and 95 % whiskers and the outliers (grey cross) of the cell or CatIB area were determined for each variant. Positive control: *E. coli* BL21(DE3) with *Ec*LDCc-Xa-SG-TDoT [9], negative control: *E. coli* BL21(DE3) with empty pET28a and wild type control: *E. coli* BL21(DE3) with pET28a::*Ec*LDCc, without any linker or aggregation inducing tag. n = 100. C_pos_ positive control, C_neg_ Negative control and LDC_WT_ wildtype *Ec*LDCc control

Microscopic images were used for comprehensive image analysis to determine the size distribution of the CatIBs and their *E. coli* producer cells (Fig. 2). The cells, carrying the CatIB plasmid with the rigid PT-Linker, were smaller, except for the TDoT and 18AWT-Tag, compared to the cells with the flexible SG-Linker (Fig. 2a; Table 1). The cells producing the *Ec*LDCc-PT-TDoT variant showed the largest cells (6.14 µm^2^) and the largest cell area distribution (1 µm^2^ to 6.14 µm^2^). The cell types producing the 18AWT variants revealed the smallest area (SG: 0.65 µm^2^, PT: 0.93 µm^2^) and the smallest median of the cell area distribution (SG: 2.01 µm^2^, PT: 2.11 µm^2^). These observations indicate that the linkers, as well as the aggregation inducing tags affect the cell morphology, i.e. size and shape.

Similar to the cell area analysis, the CatIB size analysis showed that the CatIBs seem to be smaller in combination with the PT-Linker (Fig. 2b). This time, also the TDoT variants showed the same trend. Only in combination with L6KD-Tag, the median of the distribution is similar for both linker types (PT: 0.66 µm^2^ vs. SG: 0.65 µm^2^) (Table 1). The TDoT-Tag combined with each one of the linkers revealed the smallest median of CatIB area and the smallest distribution of all variants (PT: 0.3 µm^2^ vs. SG: 0.38 µm^2^). Furthermore, *Ec*LDCc-PT-TDoT revealed the smallest CatIB area per cell area (15 %) due to large cells with small IBs. Compared to that, *Ec*LDCc-PT-L6KD showed the highest proportion of CatIB area per cell area (37 %) due to large IBs together with comparatively smaller cells. As mentioned before, no visible CatIBs were formed in combinations with 18AWT-Tag, which prohibited further morphological CatIB analysis. To conclude, the strongest influence on the CatIB area was observed for the aggregation inducing tag, while the two linkers showed quite similar data for the same aggregation inducing tag.

### Downstream processing and enzymatic activity of ten EcLDCc-CatIB variants

Besides the retained enzymatic activity of the CatIBs, a simple purification procedure, as well as a final high overall yield are important factors. In the end, the CatIB variant will be preferred that can be produced in high amounts in the cells and shows a high activity after purification. Thus, to find the best CatIB variant, not only microscopic and activity analyses, but also the CatIB purification process was included in the evaluation.

**Fig. 3:**
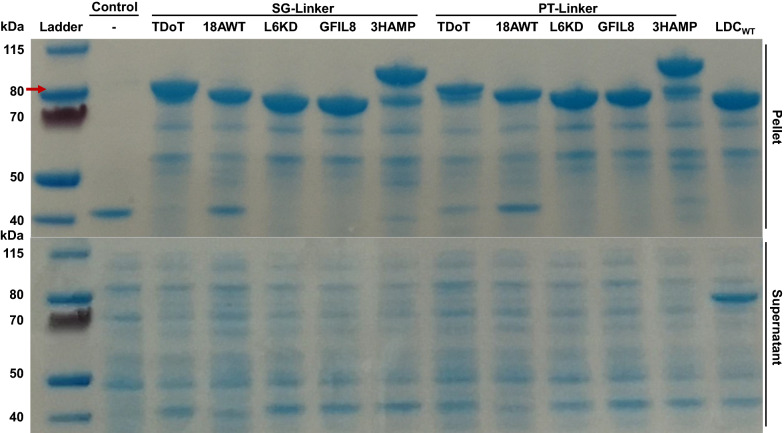
Evaluation of CatIB formation by SDS-PAGE analysis. After cultivation, the optical density of the culture were normalized to OD_600 nm_ = 12.5. The cells were disrupted and the crude cell extract was separated by centrifugation into the soluble protein containing supernatant and the insoluble CatIB‑containing pellet fractions. The pellet fraction was washed once with Milli-Q^®^ water. The samples were diluted 1:1 with SDS sample buffer and 15 µL of each sample was loaded onto the gel and stained with SimplyBlue™ SafeStain. The molecular mass of the wildtype *Ec*LDCc (81.88 kDa) is indicated by a red arrow. Molecular mass of negative control (empty pET28a): 0 kDa; *Ec*LDCc-SG-TDoT: 87.66 kDa; *Ec*LDCc-SG-18AWT: 84.22 kDa, *Ec*LDCc-SG-L6KD: 82.85 kDa, *Ec*LDCc-SG-GFIL8: 82.79 kDa, *Ec*LDCc-SG-3HAMP: 100.59 kDa, *Ec*LDCc-PT-TDoT: 88.48 kDa; *Ec*LDCc-PT-18AWT: 85.04 kDa, *Ec*LDCc-PT-L6KD: 83.67 kDa, *Ec*LDCc-PT-GFIL8: 83.61 kDa, *Ec*LDCc -PT-3HAMP: 101.41 kDa. Molecular weight determination of protein. Abbreviation: LDC_WT_: wild type *Ec*LDCc control, − Negative control (empty pET28a vector)

A previously established purification protocol [9] was simplified by using lysozyme instead of a high-pressure homogenizer for cell disruption, and testing the CatIBs directly after purification without lyophilization to enable the testing of many CatIB variants in parallel. Aliquots containing the same amount of purified CatIBs were prepared and half of these aliquots were used for the determination of CatIB weight and the other half was used for activity measurements. After purification, the production of *Ec*LDCc-CatIBs was verified by sodium dodecyl sulfate- (SDS) polyacrylamide gel electrophoresis (SDS-PAGE, Fig. 3). The SDS gel clearly shows respective bands of all *Ec*LDCc-CatIB variants only in the insoluble pellet fraction. Besides, the wild type *Ec*LDCc control showed a protein band in both the soluble and the pellet fraction. This is in good agreement with the microscopic images showing wild type *Ec*LDCc IB formation to some extent (Fig. 1). Variations in the apparent molecular mass of the different CatIB fusion proteins are due to different sizes of the aggregation inducing tags, with 3HAMP (172 aa) and TDoT (50 aa) being larger than the short tags L6KD (8 aa), 18AWT (18 aa) and GFIL8 (8 aa).

**Fig. 4:**
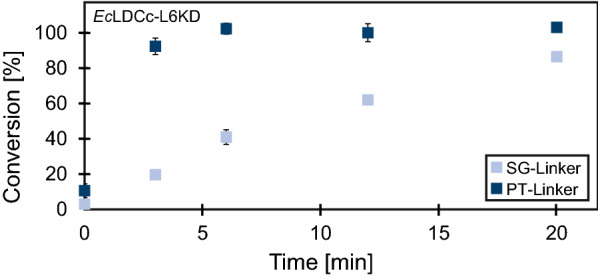
DAP production with *Ec*LDCc-L6KD variants. Experimental conditions: Purified CatIBs from cells with an OD_600 nm_ = 12.5, 10 mM l-lysine, 0.1 mM PLP in 50 mM Kpi buffer (pH 7.2). Sampling times: 0, 3, 6, 12 and 20 min. n_B_=3, except 3 min SG-L6KD (n_B_=2), and n_A_=3 (not visible, due to low standard deviation). Conversion calculated based on DAP formation from l-lysine measured by HPLC

After CatIB purification and analysis *via* SDS-PAGE, an enzymatic activity assay was performed in 50 mM Kpi buffer (pH 7.2) using l-lysine (10 mM) as a substrate and PLP (0.1 mM) as the cofactor. First, the reproducibility of the CatIB purification procedure and the enzymatic activity assay workflow were tested. To this end, three biological replicates, as well as three analytical replicates of each sampling point were sampled from *Ec*LDCc-SG-L6KD and *Ec*LDCc-PT-L6KD (Fig. 4). Activity of the CatIBs was determined by measurement of DAP formation from l-lysine over a time course of 20 minutes reaction time. Although both CatIB variants showed activity, L6KD in combination with the PT-Linker gave CatIBs with a much higher conversion rate (93 % after 3 min) compared to the SG-Linker variant (20 % after 3 min). The standard deviation between the different replicates was on average below 2.2 % for the analytical replicates and ≤ 5 % for most of the biological replicates, which proves high reproducibility of the experimental and analytical workflow procedures.

**Fig. 5:**
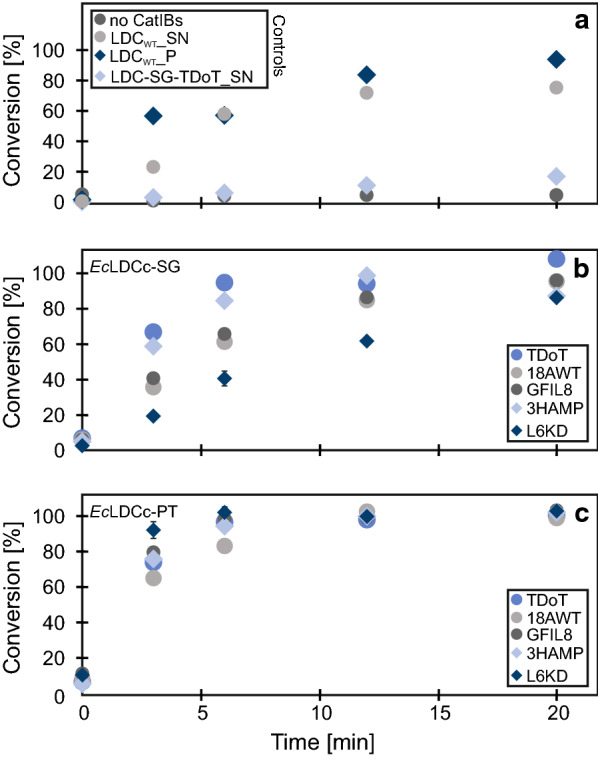
DAP production with 10 *Ec*LDCc-CatIB variants—comparison of linkers. Enzymatic activity of: **a** controls with no CatIBs, supernatant and pellet fraction of wild type *Ec*LDCc and supernatant of *Ec*LDCc-SG-TDoT **b** EcLDCc-SG variants and **c** *Ec*LDCc-PT variants. Experimental conditions: Purified CatIBs from cells with an OD_600 nm_ = 12.5, 10 mM l-lysine, 0.1 mM PLP in 50 mM Kpi buffer (pH 7.2). Sampling times: 0, 3, 6, 12 and 20 min. n_A_=3 (not visible, due to low standard deviation) and L6KD-Tag variants: n_B_=3, except 3 min SG-L6KD (n_B_=2). *P* pellet, *SN* supernatant. Conversion calculated based on DAP formation from l-lysine measured by HPLC

After having determined the reproducibility of the activity for the L6KD variants, the remaining eight CatIB constructs as well as controls were tested for their activity. As expected, the negative control, *E. coli* BL21(DE3) with an empty pET28a vector did not show any enzymatic activity (Fig. 5a; Table 1). Another control was the soluble fraction of the *Ec*LDCc-SG-TDoT CatIB producing strain. The soluble fraction showed a very low conversion of l-lysine (4 % after 3 min) meaning that a very small portion of the *Ec*LDCc was still present in the soluble fraction. Interestingly, the wild type *Ec*LDCc displayed enzymatic activity in the pellet (57 % conversion after 3 min) as well as in the supernatant fraction (24 % conversion after 3 min). Strikingly, these natural CatIBs seemed to be more active compared to the supernatant fraction of the wild type enzyme. However, the wild type *Ec*LDCc showed a smaller fraction on the SDS gel (Fig. 3), i.e., a substantial portion of the soluble protein fraction seemed to be converted into insoluble IBs during production of the recombinant protein. These natural IBs showed a higher specific volumetric productivity (specific P_v_) compared to three of the SG-Linker variants (*Ec*LDCc-SG-18AWT/L6KD/GFIL8). In comparison with all PT-Linker variants, the specific P_v_ of these natural IBs was lower (Table 1). Nevertheless, the conversion with the wild type *Ec*LDCc reached only approx. 80 % in 20 min, which might be a result of low enzyme stability, resulting in deactivation of the enzyme.

The comparison of SG-Linker variants combined with different aggregation inducing tags revealed strongly differing activities between the five CatIB constructs (Fig. 5b; Table 1). The variant with the TDoT-Tag showed the fastest conversion rate (67 % after 3 min), followed by the 3HAMP variant (59 % after 3 min), the GFIL8 variant (41 % after 3 min), the 18AWT variant (36 % after 3 min) and the L6KD variant (20 % after 3 min). In contrast, the aggregation inducing tags in combination with the more rigid PT-Linker resulted in faster conversion (65 % to 93 % after 3 min) (Fig. 5c; Table 1). Interestingly, all CatIB variants with SG-/PT-Linker showed substantial enzyme activity. However, only two SG-Linker variants reached full conversion after 12 min, while all PT-Linker variants already reached full conversion at this time point, demonstrating superior performance of all PT-Linker variants. Strikingly, the L6KD aggregation inducing tag revealed opposite results when using the SG- or the PT-Linker, respectively. While the combination of L6KD with PT shows fastest conversion of all variants, the construct with the SG-Linker resulted in the slowest conversion of all variants. This clearly demonstrates that for the investigated *Ec*LDCc CatIBs, the linker selection is a key factor of high relevance. The PT-Linker is expected to provide more rigidity than the SG-Linker and one may speculate that for the given example the linker rigidity might be an important structural aspect for the CatIB structure-function relationship.

**Fig. 6:**
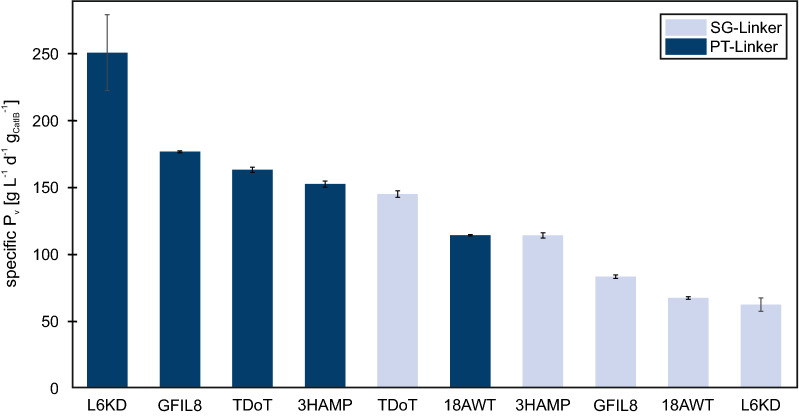
Comparison of specific volumetric productivity of ten *Ec*LDCc-CatIBs. The standard deviation of the analytical replicates are shown in black for the CatIB variants n_A_=3 (except PT-18AWT: n_A_=2). Moreover, the standard deviation of the biological duplicate/triplicate were shown in grey (PT-L6KD-Tag: n_B_=3, SG-L6KD: n_B_=2)

A general comparison of the specific P_v_s of the ten *Ec*LDCc-CatIB variants clearly illustrated that the PT-Linker led to higher specific P_v_s of the variants compared to the SG-Linker combinations (Fig. 6). Only the *Ec*LDCc-SG-TDoT variant showed a higher specific P_v_ compared to the PT variant, namely *Ec*LDCc-PT-18AWT. Furthermore, *Ec*LDCc-SG-3HAMP just revealed a slightly lower specific P_v_ of *Ec*LDCc-PT-18AWT. In both linker combinations the 18AWT-Tag showed the lowest or second to lowest specific P_v_, which makes 18AWT the weakest aggregation inducing tag of these CatIB variants. Although the activity of the 18AWT variants were low, the fact that they show substantial activity is a striking result, since visible IBs were absent in the microscopic pictures (Fig. 1) and no activity might be expected. Thus, it can be assumed that insoluble structures were formed, that were not visible under the microscope, due to potential association with parts of the cell membrane like mentioned before [28].

Comparison of the enzymatic activity data, i.e., conversion after 3 min, with the specific P_v_ of the CatIB variants led to similar results (Table 1). This gives rise to the conclusion that the applied normalization of the amount of biomass prior to purification of the different CatIBs was an effective approach to harmonize the data. Moreover, the purification efficiency seemed to be quite similar for all variants, since the optical density of the cell suspensions were normalized beforehand, and similar intensities of the protein bands were observed (Fig. 3). This indicates that there was no general distortion of the data by the purification process. There was only a small change of *Ec*LDCc-PT-3HAMP and *Ec*LDCc-PT-TDoT, with *Ec*LDCc-PT-3HAMP showing a slightly higher activity, which might be due to a slightly higher CatIB amount that was produced and purified from the cell culture. However, the most important finding can be derived from data of activity as well as specific P_v_: (i) overall the PT-Linker variants showed a higher activities and specific P_v_, (ii) the L6KD-Tag showed very different activity levels depending on the linker and (iii) 18AWT seemed to be the less suitable aggregation inducing tag for the tested system. Especially, the SG/PT-L6KD example showed that CatIBs that have the same morphology (Fig. 1) and the same amount of CatIBs per cell (Table 1) could provide very different enzymatic activity levels. The SG-Linker may result in less active CatIBs, since the portion of active enzyme inside the CatIBs could be less, or this linker may have led to a CatIB conformation that suffered from transport limitation of substrate.

### Enzymatic activity vs. morphology of EcLDCc-CatIB variants

After analyzing the microscopic images as well as the enzymatic activity of the variants, both data were combined to see if the CatIB morphology had an impact on the enzymatic activity. In case of the 18AWT-Tag an abnormal cell morphology was found. The cells did not form dense, refractive IBs, resulting in only low enzymatic activity, volumetric productivity and specific volumetric productivity (Table 1).

Three out of four PT-Linker CatIB variants, for example the CatIBs of *Ec*LDCc-PT-TDoT, showed smaller CatIB areas compared to the SG variants (PT-TDoT: 0.3 µm^2^ vs. SG-TDoT: 0.38 µm^2^). It is thus tempting to speculate that smaller IBs may retain higher enzymatic activity, possibly due to improved substrate supply to the active centers. The faster conversion rate of the PT-variants also resulted in higher P_V_s and specific P_V_s. However, in case of the TDoT variants, the enzymatic activity of both variants were in the middle range of all variants (Fig. 6). In this particular case, the size as well as the linkers seemed to have only a small impact on the enzymatic activity.

The CatIBs with the L6KD-Tags were the only ones that showed a similar size (PT: 0.66 µm^2^ vs. SG: 0.65 µm^2^), despite different linkers. Contrary to the above mentioned hypothesis, the similar morphologies of the L6KD-CatIBs were not reflected in their enzymatic activities, P_V_s nor specific P_V_s (Table 1). Whereas the combination of the SG-Linker with L6KD led to the lowest activity level (20 % conversion after 3 min), P_V_ (105 g L^− 1^ d^− 1^) and specific P_V_ (63 g L^− 1^ d^− 1^ g_CatIB_^− 1^) of all variants, the combination with the PT-Linker reached the highest activity levels (93 % after 3 min), P_V_ (457 g L^− 1^ d^− 1^) as well as specific P_V_ (256 g L^− 1^ d^− 1^ g_CatIB_^−1^). Whereas the aggregation inducing tag seemed to have a stronger effect on the CatIB and cell morphology (Fig. 1), especially, the different activity levels of L6KD showed that the impact of the linker on the activity was more pronounced.

**Table 1:** Morphological and enzymatic data of the CatIB variants and controls

	CatIBs visibility [Yes/No]	Cells with CatIBs [%]	Ø CatIBs per cell [-]	Area_M_ of CatIBs [µm²]	Area_M_ of cells [µm²]	CatIB area per cell area [%]	Pellet activity [Yes/No]	Conversion after 3 min [%]	P_v_ [g L^− 1^d^− 1^]	Specific P_v_ [g L^− 1^d^− 1^ g_CatIB_^−1^]
Control_neg_	No	0	0.00	0.00	2.72	0	No	0	0	0
LDC_WT_	Yes	88	1.35	0.47	2.94	24	Yes	57^a^ 24^b^	219^a^ 87^b^	106^a^
LDC-SG-TDoT	Yes	78	1.55	0.38	2.50	23	Yes	67	258	147
LDC-PT-TDoT	Yes	61	1.43	0.30	2.86	15	Yes	74	286	165
LDC-SG-18AWT	No	0	0.00	0.00	2.01	0	Yes	36	136	67
LDC-PT-18AWT	No	0	0.00	0.00	2.11	0	Yes	65	251	116
LDC-SG-L6KD	Yes	88	1.35	0.65	2.92	31	Yes	20	105	63
LDC-PT-L6KD	Yes	88	1.18	0.66	2.32	37	Yes	93	457	256
LDC-SG-GFIL8	Yes	72	1.44	0.49	2.76	28	Yes	41	156	83
LDC-PT-GFIL8	Yes	86	1.24	0.45	2.30	24	Yes	80	308	179
LDC-SG-3HAMP	Yes	82	1.78	0.48	2.81	32	Yes	59	227	115
LDC-PT-3HAMP	Yes	71	1.83	0.43	2.65	31	Yes	76	294	155

Pellet activity as well as conversion, after 3 min, was tested by adding 10 mM l-lysine, 0.1 mM PLP and 50 mM Kpi buffer (pH 7.2) to the CatIB pellet fraction after purification. Microscopic images were taken after main cultivation (72 h) and analyzed regarding the number of cells that carry CatIBs and the amount of CatIBs in the cells with CatIBs (n ≥ 111). Moreover, the area of cells and CatIBs were determined and the ratio of CatIB area per cell area (n = 100). Abbreviations: ^a^Pellet fraction, ^b^Supernatant fraction, SN Supernatant, Area_M_Area of the median, P_V_volumetric productivity

## Conclusions

Although there was no clear correlation between the microscopic data and enzymatic activity, the microscopic analysis is an important tool to prove the presence of IBs in the cells. Strains which do not form dense IBs and only show little cell growth, like in case of the *Ec*LDCc-18AWT variants, can be dismissed and only the strains generating clearly visible CatIB structures need to be analyzed regarding their enzymatic activity to save time and resources.

All *Ec*LDCc-CatIB variants tested showed at least some lysine decarboxylase activity. The most productive CatIB variant was L6KD in combination with the PT-Linker, showing a superior specific P_v_. However, in combination with TDoT or 3HAMP, the SG-Linker showed lower specific P_v_. Moreover, it was unexpected that the wild type *Ec*LDCc control did form natural CatIBs. However, the specific P_v_ of the natural CatIBs was much lower compared to most of the generated set of CatIBs.

Finally, it is still challenging to determine the molecular factors which led to different activities observed for different CatIBs. The analysis of the ten *Ec*LDCc-CatIB variants revealed no clear dependency on the particle size of the IBs. A more probable hypothesis could be a combination of more than one factor. For example, for the tested ten *Ec*LDCc-CatIBs it turned out that the more rigid PT-Linker resulted in CatIBs which were more active resulting in a faster conversion rate. Besides the enzymatic activity, also mass transfer could have an impact on the conversion rate. The flexible SG-Linker possibly led to denser CatIB structures that might hinder efficient substrate diffusion to the inner part of the CatIBs or may results in incorrectly folded *Ec*LDCc.

In summary, the results of our study demonstrate that for any given target enzyme the efficiency of formation and residual activity of CatIBs cannot be predicted beforehand. Thus, a large number of linkers and aggregation inducing tags need to be tested. However, the generation and testing of large CatIB libraries is time-consuming. Hence, automation of molecular biology workflows for CatIB construction, detection and activity determination are required to identify the optimal CatIB for each target enzyme.

## Methods

### Reagents and chemicals

All chemicals were purchased from ROTH and Merck (Sigma-Aldrich), unless stated otherwise. Enzymes for molecular biology were purchased from New England Biolabs GmbH (Frankfurt am Main, Germany).

### Construction of expression plasmids

The synthetic gene of the *Ec*LDCc, the two linkers, SG- and PT-Linker as well as the five aggregation inducing tags, TDoT-, 18AWT-, L6KD-, GFIL8- and 3HAMP-Tag, were synthesized by Synbio Technologies (Monmouth Junction, New Jersey, USA). The synthetic sequences contained the BsaI restriction and recognition sites, needed for Golden Gate Assembly. For Golden Gate Assembly, the synthetic gene encoding for *Ec*LDCc was assembled with one of the two linkers, one of the five aggregation inducing tags as well as the so-called suicide plasmid in a ratio of 1:1:1:3. The suicide plasmid functioned as the expression plasmid backbone. It is a pET28a vector with an integrated *ccdB* gene, coding for the CcdB toxin, which is lethal for *E. coli* DH5α and *E. coli* BL21(DE3). It served as a control for accurate Golden Gate Assembly, because of zero-background cloning [42]. During Golden Gate Assembly BsaI removed the *ccdB* gene and the T4-ligase inserted the CatIB-Linker-Tag sequence. After transformation of *E. coli* DH5α with the Golden Gate Assembly mixture only strains with the successful CatIB plasmid can grow while strains carry the original vector will be killed due to the produced toxin. Moreover, 2.5 % (v/v) T4-ligase as well as 2.5 % (v/v) BsaI restriction enzyme, were added to the mixture. The Golden Gate Assembly was performed in a PCR cycler (37 °C, 5 min and 16 °C, 5 min—cycles; 65 °C, 20 min). Information about all plasmids that were used in this study are summarized in Table S1, Additional file 1. The final expression plasmids were sequenced and verified for the correct assembly (Eurofins GmbH, Hamburg, Germany). Information about construction of the positive control strain *Ec*LDCc-Xa-SG-TDoT together with experimental characterization is provided by Kloss et al. [9].

### Protein production, cell disruption and protein purification

CatIB production was performed by cultivating *E. coli* BL21(DE3) carrying the respective expression plasmids in M9 autoinduction medium (See Table S2, Additional file 1). Following a modified protocol by Lamm et al. [43], 500 mL shaking flasks were used with a filling volume of 50 mL and a shaking frequency of 170 rpm (Infors HT Multitron Standard, Infors AG, Bottmingen, Swiss). The main cultivation was inoculated with an OD_600 nm_ of 0.05 of an overnight culture in LB complex medium (37 °C, 170 rpm). The incubation was performed in two phases. The first one was a growth phase at 37 °C for 3 h, followed by a second phase at15°C for 69 h to produce active *Ec*LDCc-CatIBs. The optical density of the main cultures was determined to perform a normalization of the cell cultures to OD_600 nm_ = 12.5. The purification process was continued with 15 mL cell suspension with the specific optical density. The cells were harvested by centrifugation at 5000 x*g* for 10 min. Another centrifugation step (3 min at 5000 x*g*) was performed after washing the cell pellet with 10 mL of 0.9 % NaCl solution. Cell lysis was performed by adding 1.35 mL cell lysis buffer, BugBuster^®^ HT Protein Extraction Reagent (Merck KGaA, Darmstadt, Germany) with the addition of 0.146 g L^− 1^ lysozyme, to the cell pellet. The suspension was incubated at 20 °C for 20 min and 750 rpm. After cell lysis the soluble and insoluble protein fraction were separated by centrifugation at 5000 x*g* for 30 min. The pellet was washed with 10 mL Milli-Q^®^ followed by centrifugation. 15 mL Milli-Q^®^ was added to the CatIB pellet and 1 mL aliquots in 1.5 mL Eppis were made. A centrifugation step was performed and the Milli-Q^®^ was discarded (10,000 x*g* for 5 min). The pellets were used for enzyme assay or for weight determination. The CatIB weight was determined via drying the pellet at 80 °C for 24 h and was then weighed. The enzymatic assay samples were stored on ice at 4 °C overnight and used for enzyme activity measurements the next day.

### Sodium dodecyl sulfate‐polyacrylamide gel electrophoresis (SDS-PAGE)

Sample preparation for SDS-PAGE analysis was performed by adding 2x Laemmli sample buffer to a purified CatIB-Milli-Q^®^ suspension originated from a cell culture with a normalized OD_600 nm_ of 12.5 (See Protein production, cell disruption and protein purification) as well as to the soluble fraction. After sample incubation for 10 min at 95 °C, the samples with the insoluble fraction were centrifuged for 5 min at 11,000 x *g*. Samples were applied to a Criterion™ 4–12 % Bis-Tris protein gel, 1.0 mm, with 18 wells (Bio-Rad Laboratories GmbH, Feldkirchen, Germany) together with a protein marker (PageRuler Prestained Protein *ladder*, ThermoFisher Scientific). Gel electrophoresis was performed in NuPAGE™ MES SDS running buffer (1×) at 200 V, 500 mA and 150 W. The gel was stained with SimplyBlue™ SafeStain. The theoretical molecular mass of the enzymes were determined by using the Protein isoelectric point calculator tool (http://isoelectric.org/).

### Determination of lysine decarboxylase activity

Enzyme activity was determined by adding 1 mL of 50 mM Kpi buffer (pH 7.2), 0.1 mM pyridoxalphosphat (PLP) and 10 mM l-lysine to the CatIB pellet, originated from a cell culture with a normalized OD_600 nm_ of 12.5 (See Protein production, cell disruption and protein purification and incubation). The soluble fraction, after cell lysis, was refilled to the normalized volume of 12 mL with Kpi-PLP-l-lysine ratio compared to the CatIB pellet fraction. 1 mL of the solution was used for the enzyme assay. The samples were incubated at 1000 rpm and 30 °C. Samples were taken after 0, 3, 6, 12 and 20 min and the enzyme was inactivated by adding 80 % (v/v) methanol and subsequently l-lysine and DAP concentrations were determined by HPLC to calculate conversion rate.

### HPLC analysis

To determine the DAP and l-lysine concentration, an amino acid HPLC system (Agilent 1260 Infinity II, Agilent Technologies, Santa Clara, USA) was used. The system was equipped with a fluorescence detector (excitation: 230 nm; emission: 450 nm) and a C18 Kinetex Evocolumn (Phenomenex, Torrence, USA). Before injection, the samples for the enzyme assay (See Activity assay) were diluted with 50 mM Kpi Buffer (pH 7.2) to a final dilution ratio of 1:500 (v/v), filtrated and then 1:1 (v/v) diluted with 100 µM α-aminobutyric acid (Sigma-Aldrich, St. Louis, USA) as the internal standard (Sigma-Aldrich, St. Louis; USA). For analyzing l-lysine and DAP concentrations in the samples, an amino acid quantification method, including a pre-column derivatisation step at 18 °C using 9 µL *ortho*-phthaldialdehyde (OPA, Sigma-Aldrich) and 1 µL of the sample (6 mixing iteration steps). The mobile phase A contained 2.63 g L^− 1^ Na_2_HPO_4_, 2.08 g L^− 1^ NaH_2_PO_4_ and 0.5 % (v/v) THF in Milli-Q^®^ water, and the mobile phase B contained 50 % (v/v) methanol, 45 % (v/v) acetonitrile, and 5 % (v/v) Milli-Q^®^ water. Chromatographic separation was performed with a linear gradient that was applied with a flow rate of 1 mL min^− 1^ (0 % B, 0–2 min 0–38 % B, 2–6 min 38–42 % B, 6–7 min 42–70 % B, 713 min 70–100 % B, 13–17 min 100-0 % B). α-Aminobutyric acid showed an approximate retention time of 6.1 min, l-lysine of 9.6 min and DAP of 11.6 min. The DAP and l-lysine concentrations were calculated with a linear calibration curve of eight reference solutions (0.5 µM to 15 µM) after normalization with the internal standard peak area (calibration curve, See Additional file 1: Fig. S1).

### Microscopic analysis

Phase-contrast microscopic analysis was performed for *E. coli* BL21(DE3) strains with CatIB formation and for control strains. Cell suspension samples from cultivation experiments were taken before CatIB purification and analyzed by microscopy. A volume of 1 µL was applied on a microscope slide and covered with a coverslip. The microscope slide was positioned upside down on the desk of an inverted Nikon Eclipse Ti2 microscope (Nikon GmbH, Düsseldorf, Germany). The sample was observed with a CFI Plan Apo Lambda 100X Oil objective (Nikon GmbH, Düsseldorf, Germany) and images were taken with a Thorlab camera DCC154M-GL (Thorlabs Inc., Newton, New Jersey, USA). Analysis of cell images were performed with Fiji ImageJ [44] to determine the areas of at least 100 inclusion bodies and cells.

## Supplementary Information

**Additional file 1.** Additional tables and figure.

## Data Availability

All data generated or analyzed during this study are included in this article and its Additional file 1.

## Abbreviations

aa: Amino acids
CatIB: Catalytically active inclusion body
DAP: 1,5-diaminopentane
GOI: Gene of interest
IB: Inclusion body
PLP: Pyridoxal-5-phosphate
P_v_: Volumetric productivity
OPA: *ortho*-phthaldialdehyde
SDS-PAGE: Sodium dodecyl sulfate polyacrylamide gel electrophoresis

## References

[1] Chung H, Yanf JE, Ha JY, Chae TU, Shin JH, Gustavsson M, Lee SY (2015). Bio-based production of monomers and polymers by metabolically engineered microorganisms. Curr Opin Biotechnol.

[2] Kremer F, Blank LM, Jones PR, Akhtar MK (2015). A comparison of the microbial production and combustion characteristics of three alcohol biofuels: Ethanol, 1-butanol, and 1-octanol. Front Bioeng Biotechnol.

[3] Wendisch VF (2014). Microbial production of amino acids and derived chemicals: Synthetic biology approaches to strain development. Curr Opin Biotechnol.

[4] Erickson B, Nelson, Winters P (2012). Perspective on opportunities in industrial biotechnology in renewable chemicals. Biotechnol J.

[5] Winnacker M, Rieger B (2016). Biobased polyamids: recent advances in basic and applied research. Macromol Rapid Commun.

[6] Thielen M (2010). Bio-polyamides for automotive applications. Bioplastics Mag.

[7] Kind S, Jeong WK, Schröder H, Wittmann C (2010). Systems-wide metabolic pathway engineering in *Corynebacterium glutamicum* for bio-based production of diaminopentane. Metab Eng.

[8] Mimitsuka T, Sawai H, Hatsu M, Yamada K (2007). Metabolic engineering of *Corynebacterium glutamicum* for cadaverine fermentation. Biosci Biotechnol Biochem.

[9] Kloss R, Limberg MH, Mackfeld U, Hahn D, Grünberger A, Jäger VD, Krauss U, Oldiges M, Pohl M (2018). Catalytically active inclusion bodies of l-lysine decarboxylase from *E. coli* for 1,5-diaminopentane production. Scientific reports.

[10] Tufvesson P, Lima-Ramos J, Nordblad M, Woodley J (2011). Guidelines and cost analysis for catalyst production in biocatalytic processes. Org Process Res Dev.

[11] Ray M, Mishra P, Das P, Sabat SC (2012). Expression and purification of soluble bio-active rice plant catalase-A from recombinant *Escherichia coli*. J Biotechnol.

[12] Zhang Y, Zborníková E, Rejman D, Gerdes K (2018). Novel (p)ppGpp binding and metabolizing proteins of *Escherichia coli*. mBio.

[13] Mathieu K, Javed W, Vallet S, Lesterlin C, Candusso MP, Ding F, Xu XN, Ebel C, Jault JM, Orelle C (2019). Functionality of membrane proteins overexpressed and purified from *E. coli* is highly dependent upon the strain. Sci Rep.

[14] Rios GM, Belleville MP, Paolucci D, Sanchez J (2004). Progress in enzymatic membrane reactors–a review. J Memb Sci.

[15] Lindeque RM, Woodley JM (2019). Reactor selection for effective continuous biocatalytic production of pharmaceuticals. Catalysts.

[16] Sheldon RA, Pereira PC (2017). Biocatalysis engineering: the big picture. Chem Soc Rev.

[17] Bornscheuer UT (2003). Immobilizing enzymes: how to create more suitable biocatalysts. Angew Chem Int Ed.

[18] Garcia-Galan C, Berenguer-Murcia A, Fernandez-Lafuente R, Rodrigues RC (2011). Potential of different enzyme immobilization strategies to improve enzyme performance. Adv Synth Catal.

[19] Datta S, Christena LR, Rajaram YRS (2013). Enzyme immobilization: an overview on techniques and support material. 3 Biotech.

[20] Sheldon RA, Schoevaart R, Van Langen LM (2005). Cross-linked enzyme aggregates (CLEAs): a novel and versatile method for enzyme immobilization (a review). Biocatal Biotransfor.

[21] Eş I, Vieira JDG, Amaral AC (2015). Principles, techniques, and applications of biocatalyst immobilization for industrial application. Appl Microbiol Biotechnol.

[22] Krauss U, Jäger VD, Diener M, Pohl M, Jaeger KE (2017). Catalytically-active inclusion bodies–carrier-free protein immobilizates for application in biotechnology and biomedicine. J Biotechnol.

[23] Jäger VD, Lamm R, Küsters K, Ölçücü G, Oldiges M, Jaeger KE, Büchs J, Krauss U (2020). Catalytically-active inclusion bodies for biotechnology-general concepts, optimization, and application. Appl Microbiol Biotechnol.

[24] Jäger VD, Kloss R, Grünberger A, Seide S, Hahn D, Karmainski T, Piqueray M, Embruch J, Longerich S, Mackfeld U, Jaeger KE, Wiechert W, Pohl M, Krauss U (2019). Tailoring the properties of (catalytically)-active inclusion body. Microb Cell Fact.

[25] Diener M, Kopka B, Pohl M, Jaeger KE, Krauss U (2016). Fusion of a coiled-coil domain facilitates the high-level production of catalytically active enzyme inclusion bodies. Chemcatchem.

[26] Zhou B, Ying L, Wu W, Zhang XE, Lin Z (2012). Small surfactant-like peptides can drive soluble proteins into active aggregates. Microb Cell Fact.

[27] Wang X, Zhou B, Hu W, Zhao Q, Lin Z (2015). Formation of active inclusion bodies induced by hydrophobic self-assembling peptide GFIL8. Microb Cell Fact.

[28] Lin Z, Zhou B, Wu W, Xing L, Zhao Q (2013). Self-assembling amphipathic alpha-helical peptides induce the formation of active protein aggregates in vivo. Faraday Discuss.

[29] Nahalka J, Nidetzky B (2007). Fusion to a pull-down domain: a novel approach of producing *Trigonopsis variabilis* D-amino acid oxidase as insoluble enzyme aggregates. Biotechnol Bioeng.

[30] Nahalka J (2008). Physiological aggregation of maltodextrin phosphorylase from *Pyrococcus furiosus* and its application in a process of batch starch degradation to α-D-glucose-1-phosphate. J Ind Microbiol Biotechnol.

[31] García-Fruitós E, González-Montalbán N, Morell M, Vera A, Ferraz RM, Arís A, Ventura S, Villaverde A (2005). Aggregation as bacterial inclusion bodies does not imply inactivation of enzymes and fluorescent proteins. Microb Cell Fact.

[32] Jiang L, Xiao W, Zhou X, Wang Q, Fan J (2019). Comparative study of the insoluble and soluble Ulp1 protease constructs as Carrier free and dependent protein immobilizates. J Biosci Bioeng.

[33] Wu W, Xing L, Zhou B, Lin Z (2011). Active protein aggregates induced by terminally attached self-assembling peptide ELK16 in *Escherichia coli*. Microb Cell Fact.

[34] Choi SL, Lee SJ, Ha JS, Song JJ, Rhee YH, Lee SG (2011). Generation of catalytic protein particles in *Escherichia coli* cells using the cellulose-binding domain from *Cellulomonas fimi* as a fusion partner. Biotechnol Bioprocess Eng.

[35] Arie JP, Miot M, Sassoon N, Betton JM (2006). Formation of active inclusion bodies in the periplasm of *Escherichia coli*. Mol Microbiol.

[36] Engler C, Kandzia R, Marillonnet SA, One Pot (2008). One step, precision cloning method with high throughput capability. PLoS ONE.

[37] Kloss R, Karmainksi T, Jäger VD, Hahn D, Grünberger A, Baumgart M, Krauss U, Jaeger KE, Wiechert W, Pohl M (2018). Tailor-made catalytically active inclusion bodies for different applications in biocatalysis. Catal Sci Technol.

[38] Rokney A, Shagan M, Kessel M, Smith Y, Rosenshine I, Oppenheim AB (2009). *E. coli* transports aggregated proteins to the poles by a specific and energy-dependent process. J Mol Biol.

[39] Rudolph R, Lilie H (1995). In vitro folding of inclusion body proteins. FASEB J.

[40] Kwon KS, Lee S, Yu MH (1995). Refolding of α_1_-antitrypsin expressed as inclusion bodies in *Escherichia coli*: characterization of aggregation. Biochim Biophys Acta.

[41] Sunitha K, Chung BH, Jang KH, Song KB, Kim CH, Rhee SK (2000). Refolding and purification of *Zymomonas mobilis* levansucrase produced as inclusion bodies in fed-batch culture of recombinant *Escherichia coli*. Protein Expr Purif.

[42] Miyazaki K (2010). Lethal *ccdB* gene-based zero-background vector for construction of shotgun libraries. J Biosci Bioeng.

[43] Lamm R, Jäger VD, Heyman B, Berg C, Gürten C, Krauss U, Jaeger K-E, Büchs J (2020). Detailed small-scale characterization and scale-up of active YFP inclusion body production with *Escherichia coli* induced by a tetrameric coiled coil domain. J Biosci Bioeng.

[44] Schneider CA, Rasband WS, Eliceiri KW (2012). NIH Image to ImageJ: 25 years of image analysis. Nat Methods.

