# Supplementary material for: Construction and comprehensive characterization of an EcLDCc-CatIB set—varying linkers and aggregation inducing tags
Source: Microb Cell Fact. 2021 Feb 17;20:49. doi: 10.1186/s12934-021-01539-w (PMC7891155; doi:10.1186/s12934-021-01539-w)
Supplement: Supplementary file 1 — Additional file 1. Additional tables and figure. [file 12934_2021_1539_MOESM1_ESM.docx]

**Additional file 1**

**“Construction and comprehensive characterization of an *Ec*LDCc-CatIB library – varying linkers and aggregation inducing tags”**

Kira Küsters^1,2^, Martina Pohl^1^, Ulrich Krauss^1,3^, Gizem Ölçücü^1,3^, Sandor Albert^1,4^, Karl-Erich Jaeger^1,3^, Wolfgang Wiechert^1,5^, Marco Oldiges^1,2*^

**Additional file Table A1:** Plasmids used in this study.

| Vector | Genotype |
| --- | --- |
| pET28a | *ColE1 lacZ’* Kan^R^ P_T7_ P_lac_ |
| pET28a::CcdB | *ColE1 lacZ’* Kan^R^ P_T7_ P_lac_ *ccdB* |
| pET28a::*Ec*LDCc::SG::TDoT | 2343 bp *Ec*LDCc::SG::TDoT fragment in pET28a |
| pET28a::*Ec*LDCc::SG::18AWT | 2247 bp *Ec*LDCc::SG::18AWT fragment in pET28a |
| pET28a::*Ec*LDCc::SG::L6KD | 2217 bp *Ec*LDCc::SG::L6KD fragment in pET28a |
| pET28a::*Ec*LDCc::SG::GFIL8 | 2217 bp *Ec*LDCc::SG::GFIL8fragment in pET28a |
| pET28a::*Ec*LDCc::SG::3HAMP | 2709 bp *Ec*LDCc::SG::3HAMP fragment in pET28a |
| pET28a::*Ec*LDCc::PT::TDoT | 2355 bp *Ec*LDCc::PT::TDoT fragment in pET28a |
| pET28a::*Ec*LDCc::PT::18AWT | 2259 bp *Ec*LDCc::PT::18AWT fragment in pET28a |
| pET28a::*Ec*LDCc::PT::L6KD | 2229 bp *Ec*LDCc::PT::L6KD fragment in pET28a |
| pET28a::*Ec*LDCc::PT::GFIL8 | 2229 bp *Ec*LDCc::PT::GFIL8 fragment in pET28a |
| pET28a::*Ec*LDCc::PT::3HAMP | 2721 bp *Ec*LDCc::PT::3HAMP fragment in pET28a |

**Additional file Table A2**: Recipe of M9 Autoinduction medium – 1000 mL

| Salt Stock solution (5x) | 200 mL |
| --- | --- |
| MgSO_4_*7H_2_O solution (246.48 g L-1) | 1 mL |
| CaCl_2_*5H_2_O solution (14.702 g L-1) | 1 mL |
| Trace element solution (1000x) | 1 mL |
| Citrate/Fe solution  (7.5 g L-1 FeSO4*7H2O  113.95 g L-1 tri-NaCitrat*2H2O) | 2 mL |
| Thiamin solution (10 g L-1) | 1 mL |
| 2 % (w/v) Lactose solution | 100 mL |
| 5 % (w/v) Glucose solution | 10 mL |
| Glycerin 99% | 4 mL |
| Kanamycin solution (50 g L-1) | 1 mL |
| add Milli-Q (final volume) | 1000 mL |
|  |  |
| **Salt Stock (5x)** | 1000 mL |
| (NH_4_)_2_SO_4_ | 25 g |
| KH_2_PO_4_ | 15 g |
| Na_2_HPO_4_ | 33.9 g |
| NaCl | 2.5 g |
| NH_4_Cl | 10 g |
| add Milli-Q (final volume) | 1000 mL |
|  |  |
| **Trace elements (1000x)** | 1000 mL |
| AlCl3*6H2O | 0.75 g |
| CoCl2*6H2O | 0.6 g |
| CuSO_4_*5H_2_O | 2.5 g |
| H_3_Bo_3_ | 0.5 g |
| MnSO_4_*1H_2_O | 17.1 g |
| Na_2_MoO_4_*2H_2_O | 3 g |
| NiCl2*6H_2_O | 1.7 g |
| ZnSO_4_*7H_2_O | 15 g |
| Dissolve in 100 mL Milli-Q and 50 mL 32% HCl and add Milli-Q to final volume | |

**Amino acid HPLC calibration curve**

**
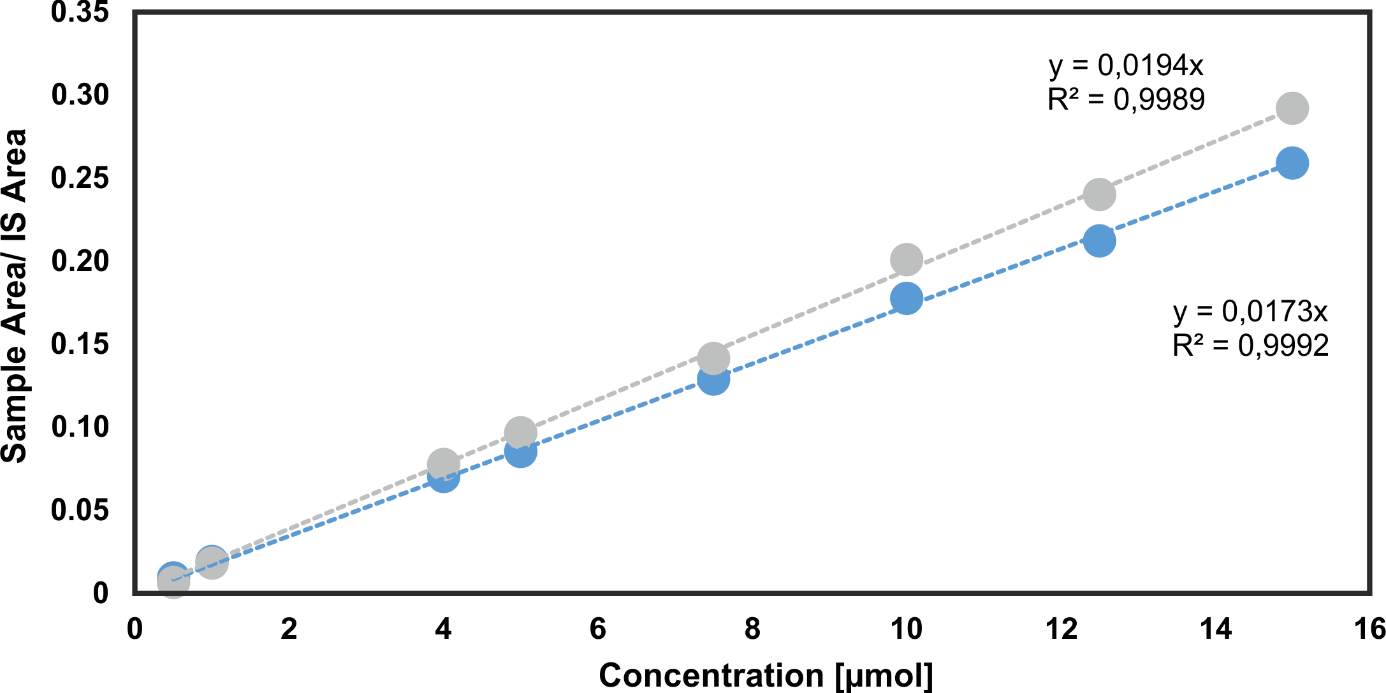
**

Additional file Figure A1: Calibration curve for DAP (grey) and l-lysine (blue) with the internal standard (IS) AABA (α-aminobutyric acid), HPLC analysis (See Methods in main paper).

**Nucleotide sequences of *Ec*LDCc, linkers and aggregation tags**

***Ec*LDCc sequence**

ATGAACATCATTGCCATTATGGGACCGCATGGCGTCTTTTATAAAGATGAGCCCATCAAAGAACTGGAGTCGGCGCTGGTGGCGCAAGGCTTTCAGATTATCTGGCCACAAAACAGCGTTGATTTGCTGAAATTTATCGAGCATAACCCTCGAATTTGCGGCGTGATTTTTGACTGGGATGAGTACAGTCTCGATTTATGTAGCGATATCAATCAGCTTAATGAATATCTCCCGCTTTATGCCTTCATCAACACCCACTCGACGATGGATGTCAGCGTGCAGGATATGCGGATGGCGCTCTGGTTTTTTGAATATGCGCTGGGGCAGGCGGAAGATATCGCCATTCGTATGCGTCAGTACACCGACGAATATCTTGATAACATTACACCGCCGTTCACGAAAGCCTTGTTTACCTACGTCAAAGAGCGGAAGTACACCTTTTGTACGCCGGGGCATATGGGCGGCACCGCATATCAAAAAAGCCCGGTTGGCTGTCTGTTTTATGATTTTTTCGGCGGGAATACTCTTAAGGCTGATGTCTCTATTTCGGTCACCGAGCTTGGTTCGTTGCTCGACCACACCGGGCCACACCTGGAAGCGGAAGAGTACATCGCGCGGACTTTTGGCGCGGAACAGAGTTATATCGTTACCAACGGAACATCGACGTCGAACAAAATTGTGGGTATGTACGCCGCGCCATCCGGCAGTACGCTGTTGATCGACCGCAATTGTCATAAATCGCTGGCGCATCTGTTGATGATGAACGATGTAGTGCCAGTCTGGCTGAAACCGACGCGTAATGCGTTGGGGATTCTTGGTGGGATCCCGCGCCGTGAATTTACTCGCGACAGCATCGAAGAGAAAGTCGCTGCTACCACGCAAGCACAATGGCCGGTTCATGCGGTGATCACCAACTCCACCTATGATGGCTTGCTCTACAACACCGACTGGATCAAACAGACGCTGGATGTCCCGTCGATTCACTTCGATTCTGCCTGGGTGCCGTACACCCATTTTCATCCGATCTACCAGGGTAAAAGTGGTATGAGCGGCGAGCGTGTTGCGGGAAAAGTGATCTTCGAAACGCAATCGACCCACAAAATGCTGGCGGCGTTATCGCAGGCTTCGCTGATCCACATTAAAGGCGAGTATGACGAAGAGGCCTTTAACGAAGCCTTTATGATGCATACCACCACCTCGCCCAGTTATCCCATTGTTGCTTCGGTTGAGACGGCGGCGGCGATGCTGCGTGGTAATCCGGGCAAACGGCTGATTAACCGTTCAGTAGAACGAGCTCTGCATTTTCGCAAAGAGGTCCAGCGGCTGCGGGAAGAGTCTGACGGTTGGTTTTTCGATATCTGGCAACCGCCGCAGGTGGATGAAGCCGAATGCTGGCCCGTTGCGCCTGGCGAACAGTGGCACGGCTTTAACGATGCGGATGCCGATCATATGTTTCTCGATCCGGTTAAAGTCACTATTTTGACACCGGGGATGGACGAGCAGGGCAATATGAGCGAGGAGGGGATCCCGGCGGCGCTGGTAGCAAAATTCCTCGACGAACGTGGGATCGTAGTAGAGAAAACCGGCCCTTATAACCTGCTGTTTCTCTTTAGTATTGGCATCGATAAAACCAAAGCAATGGGATTATTGCGTGGGTTGACGGAATTCAAACGCTCTTACGATCTCAACCTGCGGATCAAAAATATGCTACCCGATCTCTATGCAGAAGATCCCGATTTCTACCGCAATATGCGTATTCAGGATCTGGCACAAGGGATCCATAAGCTGATTCGTAAACACGATCTTCCCGGTTTGATGTTGCGGGCATTCGATACTTTGCCGGAGATGATCATGACGCCACATCAGGCATGGCAACGACAAATTAAAGGCGAAGTAGAAACCATTGCGCTGGAACAACTGGTCGGTAGAGTATCGGCAAATATGATCCTGCCTTATCCACCGGGCGTACCGCTGTTGATGCCTGGAGAAATGCTGACCAAAGAGAGCCGCACAGTACTCGATTTTCTACTGATGCTTTGTTCCGTCGGGCAACATTACCCCGGTTTTGAAACGGATATTCACGGCGCGAAACAGGACGAAGACGGCGTTTACCGCGTACGAGTCCTAAAAATGGCGGGA

**SG-Linker sequence**

AGCGGCGGTGGGTCTGGAGGCGGCTCAGGTGGTGGGTCG

**PT-Linker sequence**

CCGACCCCACCGACCACGCCAACGCCACCAACCACCCCAACCCCGACGCCG

**TDoT sequence**

ATCATTAACGAAACTGCCGATGACATCGTTTATCGCCTGACAGTCATTATCGATGATCGCTACGAATCGCTGAAAAACCTGATTACCTTACGTGCAGATCGCTTGGAGATGATCATCAATGACAATGTGTCCACCATTCTCGCGAGCATTTAA

**3HAMP sequence**

ATGGGCCTGTTTAACGCCCATGCAGTTGCGCAGCAACGCGCGGATCGCATTGCGACTCTCCTGCAGTCCTTTGCGGATGGTCAGTTGGACACCGCCGTGGGTGAAGCGCCAGCACCTGGTTACGAACGCCTGTATGACTCGCTTCGCGCCCTTCAGCGCCAACTGCGCGAACAACGTGCGGAGTTACAACAGGTTGAGAGCCTGGAAGCAGGCTTGGCTGAAATGAGTCGGCAGCATGAAGCAGGGTGGATTGACCAGACGATTCCGGCTGAACGGTTAGAGGGCCGTGCAGCACGTATCGCCAAAGGCGTGAATGAGCTGGTTGCTGCGCACATTGCGGTGAAAATGAAAGTCGTGAGCGTAGTCACCGCGTATGGCCAAGGGAACTTCGAACCGCTCATGGATCGCCTGCCGGGTAAGAAAGCCCAGATCACGGAGGCCATTGATGGCGTACGTGAACGCCTGCGTGGAGCTGCTGAAGCGACCTCTGCGCAGCTGGCCACAGCCGCCTACAATTAA

**18AWT sequence**

GAGTGGCTGAAAGCGTTCTACGAAAAGGTCCTGGAGAAACTGAAAGAACTGTTCTA

**L6KD sequence**

CTGCTGCTGCTGCTGCTGAAAGATTAA

**GFIL8 sequence**

GGTTTCATTCTGGGTTTCATTCTGTAA
